# [^18^F]FDG PET/CT Radiomics in Cervical Cancer: A Systematic Review

**DOI:** 10.3390/diagnostics15010065

**Published:** 2024-12-30

**Authors:** Judicael Hotton, Arnaud Beddok, Abdenasser Moubtakir, Dimitri Papathanassiou, David Morland

**Affiliations:** 1 Department of Surgical Oncology, Institut Godinot, 51100 Reims, France; 2 CReSTIC, UR 3804, Université de Reims Champagne-Ardenne, 51687 Reims, France; arnaud.beddok@reims.unicancer.fr (A.B.); dimitri.papathanassiou@reims.unicancer.fr (D.P.); david.morland@reims.unicancer.fr (D.M.); 3 Department of Radiation Therapy, Institut Godinot, 51100 Reims, France; 4 Department of Nuclear Medicine, Institut Godinot, 51100 Reims, France; abdenasser.moubtakir@reims.unicancer.fr

**Keywords:** radiomics, systematic review, methodological quality, locally advanced cervical cancer, PET/CT

## Abstract

**Background/Objectives**: Cervical cancer is a significant global health concern, with high incidence and mortality rates, especially in less-developed regions. [^18^F]FDG PET/CT is now indicated at various stages of management, but its analysis is essentially based on SUVmax, a measure of [^18^F]FDG uptake. Radiomics, by extracting a multitude of parameters, promises to improve the diagnostic and prognostic performance of the examination. However, studies remain heterogeneous, both in terms of patient numbers and methods, so a synthesis is needed. **Methods**: This systematic review was conducted following PRISMA-P guidelines and registered in PROSPERO (CRD42024584123). Eligible studies on PET/CT radiomics in cervical cancer were identified through PubMed and Scopus and assessed for quality using the Radiomics Quality Score (RQS v2.0), with data extraction focusing on study design, population characteristics, radiomic methods, and model performances. **Results**: The review identified 22 studies on radiomics in cervical cancer, 19 of which focused specifically on locally advanced cervical cancer (LACC) and assessed various clinical outcomes, such as survival, relapse, treatment response, and lymph node involvement prediction. They reported significant associations between prognostic indicators and radiomic features, indicating the potential of radiomics to improve the predictive accuracy for patient outcomes in LACC; however, the overall quality of the studies was relatively moderate, with a median RQS of 12/36. **Conclusions**: While radiomic analysis in cervical cancer presents promising opportunities for survival prediction and personalized care, further well-designed studies are essential to provide stronger evidence for its clinical utility.

## 1. Introduction

Cervical cancer is a major cause of death, ranking as the fourth most common cancer in women worldwide. In 2020, there were an estimated 604,000 new cases and 342,000 deaths globally, with the majority occurring in less-developed countries [1,2]. These numbers have been rising for over a decade [3]. Nearly half of patients are diagnosed at a locally advanced stage of the disease which, according to the International Federation of Gynecology and Obstetrics (FIGO) classification, includes stages IB2-IVA [4]. In locally advanced cervical cancer (LACC), standard of care is based on concurrent chemoradiotherapy (CCRT) followed by image-guided adaptative brachytherapy (IGABT) [5], with expected cure rates of 30–90% depending on prognostic factors including FIGO stage, histology, and lymph node metastases. Nevertheless, in spite of a high-complexity treatment, global survival at 5 years is estimated at around 65% [6], and one-third of patients relapse within the first 2 years of treatment [7].

Fluorine-18-labeled fluorodeoxyglucose positron emission topography associated with computed tomography ([^18^F]FDG PET/CT) is widely used in oncology as a functional imaging technique. [^18^F]FDG PET/CT has a well-established role in managing patients with cervical cancer, particularly in staging [8,9], treatment response assessment, and recurrence detection [10]. Interpretation is based mainly on qualitative visual analysis, but some semi-quantitative PET/CT-derived indexes have shown added value. Among them, the maximal [^18^F]FDG uptake value (maximal standardized uptake value—SUVmax) could help predict overall survival, treatment response, and lymph node involvement [11,12]. Volumetric parameters such as the metabolic tumor volume (MTV) or the total lesion glycolysis (TLG, product of SUV and MTV) have also been reported to be useful for the characterization of the tumor and to be significant predictors of relapse-free (RFS) and overall survival (OS) [13,14,15].

The use of more advanced PET/CT indexes, based on texture analysis, was quickly suggested as a way of improving prediction models. Radiomics, a rapidly emerging field, leverages advanced image processing techniques to enhance the predictive power of traditional imaging modalities such as PET/CT. Radiomics encompasses the extraction and quantification of numerous imaging features, often invisible to the naked eye. By analogy with genomics, radiomics aims to generate reproducible and clinically meaningful quantitative biomarkers [16]. These radiomic features can then be correlated with various clinical outcomes, including tumor characterization, treatment response, and patient survival [16]. In oncology, radiomics has attracted increasing interest due to its potential to create non-invasive predictive models that support personalized treatment approaches.

Radiomics requires, however, a robust workflow based on standardized, rigorous methods to ensure the reliability and generalizability of results [17,18]. Using the Radiomics Quality Score (RQS v2.0) and a scoring system proposed by Lambin et al. [19], designed to assess the crucial steps in radiomics pipelines, previous investigations have found that radiomics studies are heterogeneous in terms of quality.

The aim of this study was to conduct a systematic review providing an overview of the applications of [^18^F]FDG PET/CT radiomics imaging in the management of cervical cancer, with a specific focus on assessing their methodological quality using the RQS.

## 2. Materials and Methods

This systematic review of the published literature was performed according to the reporting standard of the PRISMA-P statement [20]. The review protocol was registered on the International Prospero Register of Systematic Reviews (PROSPERO, registration number: CRD42024584123).

### 2.1. Search Strategy, Eligibility Criteria

We performed a literature search in the PubMed and Scopus databases to identify all eligible articles using the following formula: (“PET” OR “positrons” OR “positron”) AND (“cervical” OR “cervix”) AND (“cancer” OR “neoplasm” OR “tumor”) AND (“radiomics” OR “radiomic” OR “texture” OR “textural”). The search formula was applied to titles only to ensure the inclusion of studies explicitly focused on [^18^F]FDG PET/CT radiomics in cervical cancer.

Results were admitted from 1 January 2000 to 31 January 2024. Reviews were automatically identified using the article type options and removed from the extracted database.

Inclusion criteria were as follows: studies based on human data, studies specifying cervical tumor type, and studies performing radiomics on PET/CT imaging. Exclusion criteria were as follows: studies not related to medical topics, reviews, erratum or editorials, cases reports, duplicates, studies outside the oncological field or radiomics not performed on PET/CT, technical articles without a clinically oriented question, studies not in English, and full text not available.

### 2.2. Data Collection

Two investigators extracted data from each selected study and reported it as follows: first author, publication year, study design; characteristics of study population: number of patients included, type of cervical cancer, type of treatment, imaging modalities; radiomics details like segmentation method and software used, radiomic feature extraction software and method, number of radiomic features extracted, type of radiomic feature extracted, type of models constructed and performance, number of radiomic/non-radiomic features included in each models, endpoints of the studies, internal and/or external validation cohorts.

### 2.3. Textural Parameters Used

The number of textural parameters that can be extracted from an image is huge, and parameters were grouped into several categories [21], which were evaluated to understand their significance and impact:

- Shape features are purely geometric descriptions of the content of the region of interest (ROI) such as sphericity and volume.
- First-order features (such as SUVmean and SUVmax) are based on each voxel’s value within the ROI, independent of spatial relationships; they are calculated directly on the image matrix.
- Second-order features quantify the spatial relationships between neighboring voxels and are computed from texture matrices, such as the gray-level co-occurrence matrix (GLCM) or the gray-level run-length matrix (GLRLM), calculated from the segmented ROI.
- Higher-order features are derived after applying mathematical transformations to the image, such as wavelet transforms, Fourier transforms, or graph-based approaches, providing multi-scale information.

### 2.4. Assessment of the Quality of the Radiomics Studies

Studies were assessed for quality based on the Radiomics Quality Score [19]. The RQS (v2.0) scores a total of 16 items divided into criteria that examine critical components of the radiomics workflow, including imaging protocols quality, radiomics index selection and validation, model performance indices, biological and/or clinical validation of the model and its usefulness, level of study evidence, and free and open access to the model and the data used to build it. The total ranges from −8 to 36. This score was already used to determine the risk of bias and the applicability of each included study. RQS was assessed by three raters, namely one surgeon (JH), one radiation therapist (AB), and one nuclear medicine physician (DM), all with several years of experience in radiomics. A consensus was reached among the raters, and the scores presented in the article reflect the agreed-upon consensus ratings.

## 3. Results

### 3.1. Literature Research

**Figure 1:**
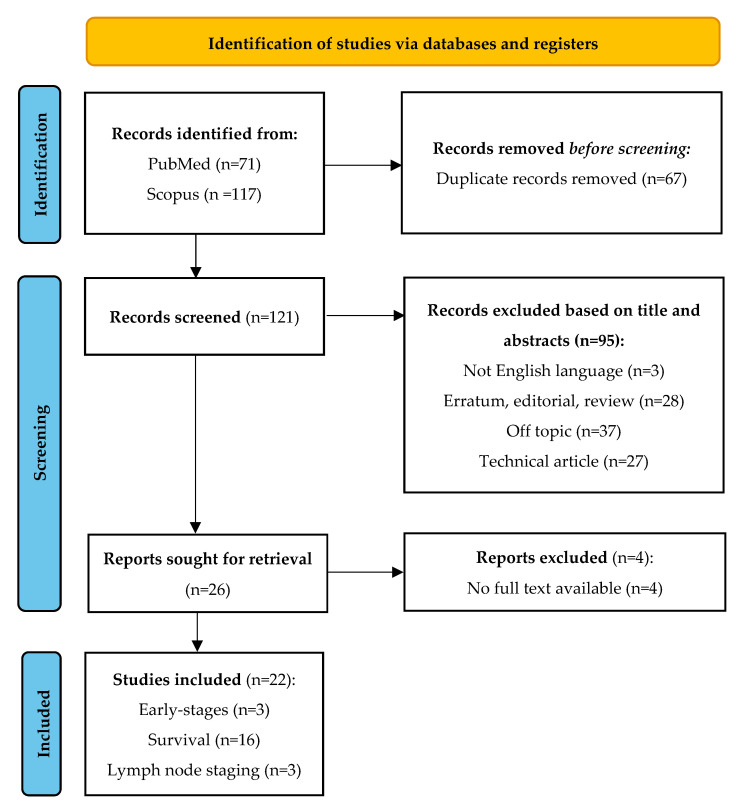
Flowchart of literature search and article selection.

The literature search yielded 188 articles. After excluding duplicates (67) and gray literature, including erratum, editorials, and reviews (28), off-topic articles (37), technical articles (27), or articles not written in English (3), and after reviewing the abstracts of the remaining articles, 26 reports were retained. Of these, 22 met the inclusion criteria (Figure 1). The remaining four articles had no full text available.

### 3.2. Qualitative Synthesis of Included Studies

Twenty-one out of twenty-two studies were retrospective. The mean number of patients included was 129 (range: 44 to 376), with 13/22 (59.1%) including more than 100 patients. The results are presented in Table 1.

#### 3.2.1. Late-Stage Cervical Cancers

Of the selected studies, 19 focused on LACC and studied the association of [^18^F]FDG radiomics on 29 outcomes: overall survival (7/29) [22,25,26,28,35,37,38], recurrence (recurrence or disease-free and progression-free survival: 12/29) [23,24,26,27,33,34,35,36,37,38,39,40], response to treatment (4/29) [24,28,42,43], lymph node involvement association (2/29) [32,41], or histology prediction (1/29) [41]. The significant radiomics parameters identified are presented in Table 1 with their corresponding matrices. For overall survival, three types of parameters were reported at least three times: SUV-based parameters, size-based parameters, and TLG. All these parameters are derived directly from the PET image, without any additional matrix calculation. For recurrence, the most frequently reported parameters were the same (size, SUV, TLG) plus the shape-parameter sphericity (three times) and gray-level non-uniformity (GLNU) derived from the gray-level run length matrix (GLRLM). In the two studies looking at the association of radiomics with lymph node involvement [32,41], size is reported (either via TLG or the largest 2D diameter).

Radiomics is most often measured on one single PET/CT, but two studies have investigated it over several time points, enabling assessment of the value of monitoring parameters over time [24,28].

#### 3.2.2. Early-Stage Cervical Cancers

In ESCC (FIGO stages IA to IIA), an association between radiomics and lymph node involvement was reported [29]. Radiomics was also linked to E-cadherin expression and disease-free survival [30,31]. Given the small number of studies, none of the radiomics parameters was cited in more than one article.

### 3.3. RQS Assessment

The RQS was calculated for each article included in the review (Supplementary Table S1). The median score, after consensus, was 12 (5–19), corresponding to a percentage of 25% (12.9–52.8). Only one of the studies was prospective. Imaging protocols were usually well detailed. Few studies (4/22) performed multiple segmentations to assess the stability of radiomics features. None conducted a phantom study. The software used often varied between studies, with authors using semi-automatic image segmentation software in 40.9% of cases (9/22). Internal validation was present in 14 out of 22 studies and external cohort validation in 5 out of 22, while 8 studies had no validation cohort. Assessment of the clinical utility of radiomic models was associated with most studies (18/22). No study performed a cost-effectiveness analysis. Likewise, none of the selected studies made their code or data publicly available.

## 4. Discussion

This article reviews the studies identified through a systematic search of PubMed and Scopus databases that focus on [^18^F]FDG radiomics in cervical cancer. The studies have mainly focused on LACC (19/22), while the analysis of ESCC is more limited, for reasons that may be technical (insufficient number of voxels to perform texture studies) or clinical (no indication for [^18^F]FDG PET/CT, no management issues). Analyses of ESCCs focus mainly on histological correlations or staging.

For LACC, studies have focused on the following domains: overall survival, prediction of disease progression, response to treatment and, more rarely, prediction of lymph node involvement. As for overall survival, the results are heterogeneous, with most of the radiomics parameters identified as relevant appearing in only one publication at a time. SUV and volume (or the combination of the two via TLG) were the exceptions. This result is in good agreement with the rest of the non-radiomics literature, which has already reported it extensively [11,12,13,14,15]. More complex textural parameters thus seem to have a lower added value. The same is true when looking for with lymph node involvement prediction, although limited evidence is available (two articles).

Textural features seem, however, promising for the prediction of disease progression. Although size and SUV parameters remain reported (again in agreement with the literature [11,12,13,14,15]), one parameter seems to be more important than others: the GLNU calculated on the GLRLM matrix. GLRLM represents the size of homogeneous runs for each gray level and GLNU_GLRLM the similarity of gray levels in the image. This parameter represents one possible way of measuring tumor heterogeneity. In the validation study by Lucia et al. [34], the GLNU_GLRLM had an AUC of 72% [64–79%] for the prediction of disease-free survival and 88% [81–93%] for the prediction of locoregional control in the training cohort. This parameter was introduced into a model also including MRI textural parameters and successfully validated on independent cohorts (N = 50 and N = 28). Flow diagrams of risk suggest that GLNU_GLRM is linked to the type of recurrence (locoregional or distant metastatic). GLNU_GLRLM was also included in the models presented by Nakajo et al. [36] (AUC for disease progression: 87.2%) and Pedraza et al. [38]. A high GLNU_GLRLM value is associated with a poorer prognosis.

Reproducibility in radiomics is significantly affected by variations in segmentation methods, particularly in PET/CT imaging. These variations can substantially alter extracted shape features, while first- and second-order features are typically less influenced, making accurate reproduction of results challenging [44]. Current recommendations suggest using (semi)automated segmentation methods wherever possible, avoiding basic fixed thresholding, and potentially employing a consensus approach to identify the most appropriate algorithm for a given image [45]. The variability in PET/CT imaging techniques, including differences in voxel size, reconstruction algorithms, and post-processing filters, can significantly impact radiomic feature extraction and repeatability. These factors highlight the importance of standardization and harmonization efforts to enhance reproducibility across studies. To develop more generalizable models and enhance the statistical relevance of findings, it is crucial to use larger, multicenter patient cohorts. Radiomics is highly dependent on factors like voxel size and reconstruction post-filtering [46]. This limitation affects the external validity of proposed models. Overall quality of studies remains low, as demonstrated by the RQS (average: 12/36). Studies published have been often monocentric (unlikely to be generalizable to other sites), retrospective (selection bias), and use cohorts with sizes between 50 and 150 patients (risks of overfitting for training models, difficult to rigorously cross-validate) [47]. One of the main limitations of the reviewed studies is the limited use of external validation cohorts. Confirmation of results on validation cohorts is inconsistent, with the exception of the Lucia et al. study [34], validated in a separate article. Only 5 out of 22 studies performed external validation, which significantly impacts the robustness and generalizability of the reported radiomic predictors. This highlights the need for future research to prioritize external validation to strengthen the clinical applicability of radiomic models.

The fact that the RQS analysis was carried out by three people with expertise in radiomics is a strength of this study. Indeed, this score for evaluating the quality of radiomic analyses is validated but requires an in-depth evaluation of the methodology of each article. The articles were read by two persons, limiting the risk of error in data collection. The main limitations of this systematic review are heterogeneity between studies, making direct comparison of results difficult. Variable methodological quality among the included studies also impacts the reliability of conclusions [48]. The reviewed studies exhibit significant heterogeneity in sample sizes, imaging techniques, and research designs, which complicates direct comparisons and consistency analysis. A formal heterogeneity analysis could provide deeper insights into the reliability and generalizability of radiomic predictors across investigations. Future meta-analyses should consider incorporating such analyses to better evaluate the robustness and consistency of radiomic features in varying clinical and technical contexts.

Another notable limitation of the included studies is the absence of open-access data. None of the reviewed studies released their datasets publicly, which significantly impacts the repeatability of their findings and hinders future validation efforts by other researchers. The lack of shared data restricts the reproducibility of radiomic workflows, limits the generalizability of results, and reduces opportunities for collaboration and innovation. Moving forward, adopting open science practices, including data sharing, is essential to improve transparency and accelerate progress in radiomics research. While 19 out of the 22 included studies focus on LACC, the limited number of studies on ESCC presents an important area for consideration. However, their limited scope and lack of consistent radiomic features highlight the challenges in applying radiomics to early-stage cases. This underscores the need for future research to address these gaps and improve the understanding and utility of radiomics in ESCC.

## 5. Conclusions

While radiomic analysis in cervical cancer presents promising opportunities for survival prediction and personalized care, further well-designed studies are essential to provide stronger evidence for its clinical utility. Standard PET/CT parameters such as volume and SUV, along with GLNU_GLRLM, seem the most promising.

## Figures and Tables

**Table 1:** Main characteristics of the selected studies.

Authors, Ref	Year	Study Design	Sample Size	Stage	Radiomic Software	Internal Valid.	External Valid.	Outcome Measures	Significant Radiomic Matrices and Corresponding Features	RQS
Alencar et al. [22]	2022	Retrospective	50	LACC	LIFEx	No	No	Overall Survival	Image (SUVpeak) GLRLM (LRLGE)	9 (25%)
Altazi et al. [23]	2018	Retrospective	80	LACC	In-house software	Yes	No	Recurrence (Loco-regional)	GLCM (Difference entropy) GLSZM (SAE, SZV, IV, LIE)	12 (33.3%)
								Recurrence (distant metastasis)	Image (Volume, Surface, Asphericity, Spherical disproportion) NGTDM (coarseness, strength)	
Burchardt et al. [24]	2023	Retrospective	83	LACC	MatLab	No	No	Response to treatment,	Image (Entropy, Coefficient of Variation, Kurtosis) GLCM (Energy, Homogeneity, Correlation)	5 (13.9%)
								Recurrence (All sites)	Image (Coefficient of Variation, Entropy)	
Carlini et al. [25]	2022	Retrospective	85	LACC	PyRadiomics	Yes	No	Overall Survival	Image (Max2DDiamSlice, SurfaceVolumeRatio, Maximum, 10Percentile)	16 (44.4%)
Chen et al. [26]	2018	Retrospective	142	LACC	PyRadiomics	Yes	No	Overall Survival	GLRLM (HGRE)	7 (19.4%)
								Recurrence (All sites)	GLRLM (HGRE)	
Ferreira et al. [27]	2021	Retrospective	158	LACC	OncoRadiomics	Yes	Yes	Disease-Free Survival	GLDZM (DZNN, DZV) GLSZM (HILAE)	19 (52.8%)
Ho et al. [28]	2016	Prospective	44	LACC	MatLab	No	No	Response to treatment	Image (SUVmax, MTV, TLG) GLRLM (SRE, LRE, RLNU, GLNU) GLSZM (LGZE, GLNU)	14 (38.9%)
								Overall Survival	Image (TLG) GLRLM (RLNU, GLNU)	
Li Kexin et al. [29]	2018	Retrospective	94	ESCC	AK software	Yes	No	Staging (LN association)	Image (TLG, skewness)	16 (44.4%)
Li et al. [30]	2021	Retrospective	110	ESCC	AK software	Yes	No	E-cadherin expression	Image (Correlation, Compactness) GLCM (Energy, Entropy) GLRLM (HGRE, LRE) GLSZM (LIE) Others (non-standard nomenclature)	13 (36.1%)
Liu et al. [31]	2022	Retrospective	201	ESCC	PyRadiomics	Yes	No	Disease-Free Survival	GLCM (SumSquares)	9 (25%)
Lucia et al. [32]	2023	Retrospective	239	LACC	Radiomics ToolBox	Yes	Yes	Staging (LN association)	Image (maxDiameter2D) GLDZM (HISDE)	12 (33.3%)
Lucia et al. [33]	2018	Retrospective	102	LACC	Not specified—IBSI compliant	Yes	No	Locoregional Control	Image (SUV via post-treatment metabolic response) GLRLM (GLNU)	14 (38.9%)
								Disease-Free Survival	Image (SUV via post-treatment metabolic response) GLRLM (GLNU)	
Lucia et al. [34]	2019	Retrospective	190	LACC	Not specified—IBSI compliant	Yes	Yes	Locoregional Control	GLRLM (GLNU)	18 (50%)
								Disease-Free Survival	GLRLM (GLNU)	
Mu et al. [35]	2020	Retrospective	154	LACC	MatLab	Yes	Yes	Overall Survival	Tumor: Image (Short Axis Diameter) Habitat: Image (Short axis diameter), Co-occurrence PET/CT (correlation)	15 (41.7%)
								Progression-Free Survival	Tumor: Image (Long Diameter); Habitat: Co-occurrence PET/CT (correlation), Spectrum (microdiagonal)	
Nakajo et al. [36]	2022	Retrospective	50	LACC	LIFEx	Yes	No	Progression-Free Survival	Image (Surface, MTV) GLRLM (RLNU, GLNU)	14 (38.9%)
Niyoteka et al. [37]	2022	Retrospective	376	LACC	PyRadiomics	Yes	Yes	Overall Survival	Image (TLG, minorAxis, Surface, Volume, LeastAxis, maximum2DDiameter, Variance, Energy) GLCM (Correlation, Joint Entropy) GLDM (Entropy, Non-uniformity) GLSZM (Entropy) GLRLM (Entropy) NGTDM (Coarseness)	17 (47.2%)
								Progression-Free Survival	Image (TLG, MinorAxis, Surface, Volume, LeastAxis, MaximumDiameter, SurfaceVolumeRatio, Variance, Energy) GLCM (Correlation, Joint Entropy) GLDM (Entropy, non-uniformity) GLSZM (Entropy, SAE) GLRLM (Entropy)	
Pedraza et al. [38]	2022	Retrospective	116	LACC	LIFEx	No	No	Overall Survival	Image (TLG, MTV, Sphericity, Compacity) GLRLM (GLNU) NGLDM (Coarseness) GLZLM (GLNU, ZLNU)	5 (13.9%)
								Recurrence-Free Survival	Image (TLG, MTV, Sphericity, Compacity) GLRLM (GLNU) GLZLM (GLNU, ZLNU)	
Reuzé et al. [39]	2017	Retrospective	118	LACC	LIFEx	Yes	No	Recurrence (all sites)	Image (SUVmean, SUVmax, SUVpeak, MTV, TLG, Entropy), GLSZM (LGZE, HGZE)	15 (41.7%)
Schernberg et al. [40]	2018	Retrospective	108	LACC	LIFEx	Yes	No	Locoregional control	Image (SUVpeak)	10 (27.8%)
Shen et al. [41]	2017	Retrospective	170	LACC	NS	Yes	No	Staging (LN association)	Image (TLG) GLCM (Homogeneity)	11 (30.6%)
								Histological type	GLSZM (SZE)	
Yang et al. [42]	2016	Retrospective	90	LACC	In-house software	Yes	No	Response to treatment	Image (Heterogeneity) GLCM (Entropy, Energy) GLZSM (GLNU, ZSNU)	5 (12.9%)
Zhou et al. [43]	2020	Retrospective	75	LACC	MatLab	Yes	No	Response to treatment	Image (SUVmin, SUVmedian, SUVsd, SUVvariance, SUVkurtosis, Orientation, Perimeter, MTV, TLG) GLCM (Energy, Entropy, Correlation, Contrast, Variance, Sum mean, Inertia, Cluster shade, Tendency, Inverse Variance)	7 (19.4%)

SUV, standardized uptake value; RQS, radiomic quality score; LACC, locally advanced cervical cancer; GLRLM, gray-level run length matrix; LRLGE, long-run low gray-level emphasis; SAE, short-are emphasis; SZV, size-zone variability; IV, intensity variance; LIE, low intensity emphasis; NGTDM, neighboring gray-tone difference matrix; GLCM, gray-level co-occurrence matrix; HGRE, high gray-level run emphasis; GLDZM, gray-level dependence zone matrix; DZNN, distance zone non-uniformity normalized; DZV, distance zone variance; GLSZM, gray-level size zone matrix; HILAE, high intensity large area emphasis; MTV, metabolic tumor volume; TLG, total lesion glycolysis; SRE, short-run emphasis; LRE, long-run emphasis; RLNU, run length non-uniformity; GLNU, gray-level non-uniformity; LGZE, low gray-level zone emphasis; ESCC, early-stage cervical cancer; LN, lymph node; HISDE, high intensity small distance emphasis; GLDM, gray-level dependence matrix; NGLDM, neighboring gray-level dependences matrix; GLZLM, gray-level zone length matrix; ZLNU, zone length non-uniformity; HGZE, high gray-level zone emphasis; NS, not specified; SZE, size zone emphasis.

## Data Availability

The original contributions presented in the study are included in the article/Supplementary Materials, further inquiries can be directed to the corresponding author.

## Supplementary Materials

The following supporting information can be downloaded at: https://www.mdpi.com/article/10.3390/diagnostics15010065/s1, Table S1: Details of radiomic quality scores for all included studies.
