# Supplementary material for: [18F]FDG PET/CT Radiomics in Cervical Cancer: A Systematic Review
Source: Diagnostics (Basel). 2024 Dec 30;15(1):65. doi: 10.3390/diagnostics15010065 (PMC11720459; doi:10.3390/diagnostics15010065)
Supplement: Supplementary file 1 [file diagnostics-15-00065-s001.zip › diagnostics-3311179-supplementary.pdf]

Supplementary Table S1. Details of radiomic quality scores for all included studies.

Study ID	Image protocol quality	Multiple segment.	Phantom study	Imag. at multiple time pts	Features reduct. or adjustment	Multi-variable analysis	Biol. correlates	Cut-off analysis	Discrim. statistics	Calibrat. statistics	Prospect. study	Valid.	Compar. to 'gold standard'	Potential clinical applic.	Cost-effectiv. analysis	Open science & data	Total points
	**0, 1, 2**	**0 – 1**	**0 – 1**	**0 – 1**	**-3 – 3**	**0 – 1**	**0 – 1**	**0 – 1**	**0, 1, 2**	**0, 1, 2**	**0 – 7**	**-5 to 5**	**0 – 2**	**0 – 2**	**0 – 1**	**0 to 4**	**-12 – 36**
**Alencar et al, [22]**	2	0	0	0	-3	0	0	1	1	0	0	-5	2	2	0	0	**9**
**Altazi et al, [23]**	1	0	0	0	3	1	0	0	1	1	0	2	2	2	0	0	**12**
**Burchardt et al, [24]**	0	0	0	0	-3	0	0	0	0	0	0	-5	2	2	0	0	**5**
**Carlini et al, [25]**	1	1	0	0	3	1	0	0	2	2	0	-5	0	2	0	0	**9**
**Chen et al, [26]**	2	0	0	0	-3	0	0	0	1	0	0	2	0	2	0	0	**7**
**Ferreira et al, [27]**	2	1	0	0	3	1	0	0	2	2	0	5	2	2	0	0	**19**
**Ho et al, [28]**	2	0	0	1	-3	0	0	1	1	0	7	-5	0	2	0	0	**14**
**Li Kexin et al, [29]**	2	0	0	0	-3	0	1	0	1	0	0	2	0	2	0	0	**16**
**Li et al, [30]**	2	0	0	0	-3	0	1	0	1	1	0	2	0	0	0	0	**13**
**Liu et al, [31]**	2	0	0	0	3	1	1	0	0	0	0	2	0	0	0	0	**9**
**Lucia et al, [32]**	2	0	0	0	3	1	0	1	1	1	0	4	0	2	0	0	**12**
**Lucia et al, [33]**	2	0	0	1	3	1	0	0	1	0	0	2	2	2	0	0	**14**
**Lucia et al, [34]**	2	0	0	1	3	1	0	1	1	0	0	4	2	2	0	0	**18**
**Mu et al, [35]**	2	0	0	0	3	1	0	1	2	1	0	3	0	2	0	0	**15**
**Nakajo et al, [36]**	2	1	0	0	3	1	0	1	2	0	0	2	0	2	0	0	**14**
**Niyoteka et al, [37]**	2	0	0	1	3	1	1	0	1	1	0	5	2	2	0	0	**17**
**Pedraza et al, [38]**	1	0	0	1	-3	0	0	0	0	0	0	-5	0	2	0	0	**5**
**Reuzé et al, [39]**	2	0	0	0	3	1	0	1	2	2	0	-5	2	2	0	0	**15**
**Schernberg et al, [40]**	2	0	0	1	-3	0	1	1	1	0	0	2	0	0	0	0	**10**
**Shen et al, [41]**	1	0	0	0	3	1	1	0	1	0	0	2	0	2	0	0	**11**
**Yang et al, [42]**	1	0	0	1	-3	0	0	0	1	0	0	-5	0	2	0	0	**5**
**Zhou et al, [43]**	1	0	0	0	3	1	0	0	1	0	0	-5	0	0	0	0	**7**
**Median scores**	**2**	**0**	**0**	**0**	**3**	**1**	**0**	**0**	**1**	**0**	**0**	**2**	**0**	**2**	**0**	**0**	**12**
